# Prevalence of Informal Caregiving in States Participating in the US Patient Protection and Affordable Care Act Balancing Incentive Program, 2011-2018

**DOI:** 10.1001/jamanetworkopen.2020.25833

**Published:** 2020-12-15

**Authors:** Rebecca Anastos-Wallen, Rachel M. Werner, Paula Chatterjee

**Affiliations:** 1 Perelman School of Medicine, Department of Medicine, University of Pennsylvania, Philadelphia; 2 Hospital of the University of Pennsylvania, Philadelphia; 3 Leonard Davis Institute for Health Economics, University of Pennsylvania, Philadelphia; 4 Corporal Michael J. Crescenz Veterans Affairs Medical Center, Philadelphia, Pennsylvania

## Abstract

**Importance:**

The Balancing Incentives Program (BIP), established under the 2010 Patient Protection and Affordable Care Act provided federal funding for states to shift long-term care out of institutional settings and into the home. However, the association of its implementation with informal caregiving is not known.

**Objective:**

To evaluate the association between BIP participation and the prevalence and frequency of informal caregiving and socioeconomic disparities among caregivers.

**Design, Setting, and Participants:**

The cohort study included respondents to the 2011-2018 American Time Use Survey in BIP-adopting states and non–BIP-adopting states.

**Exposure:**

Living in a state that had implemented the BIP after program implementation had begun (April 2012 to April 2018).

**Main Outcomes and Measures:**

Prevalence of caregiving among all respondents, frequency of caregiving, and minutes of daily sleep, a marker of well-being. Differences-in-differences (DID) regression analysis was used to compare these outcomes between BIP-adopting states and non–BIP-adopting states.

**Results:**

The study included 38 343 respondents in BIP-adopting states (median age, 47 years [interquartile range (IQR), 31-61 years]; 51.9% women), of whom 7428 were caregivers (median age, 51 years [IQR, 37-61 years]; 55.6% women), and 26 437 respondents in non–BIP-adopting states (median age, 48 years [IQR, 32-62 years]; 52.7% women), of whom 5527 were caregivers (median age, 52 years [IQR, 38-62 years]; 57.9% women). There was no change in the prevalence of caregiving between BIP-adopting and non–BIP-adopting states after program implementation (DID, 0.00%; 95% CI, −0.01% to 0.01%). Caregivers in BIP-adopting states were more likely to provide daily care after implementation (DID, 3.2%; 95% CI, 0.3%-6.0%; *P* = .03) and report increased time sleeping (DID, 15.6 minutes; 95% CI, 4.9-26.2 minutes; *P* = .005) compared with caregivers in non–BIP-adopting states. This association was more pronounced among caregivers with more education (DID, 25.1 minutes; 95% CI, 6.5-43.8 minutes; *P* = .01) and higher annual family income (DID, 16.9 minutes; 95% CI, 5.9-27.9 minutes; *P* = .004) compared with caregivers in non–BIP-adopting states who had the same education and income levels, respectively.

**Conclusions and Relevance:**

In this cohort study, the BIP was associated with increased daily caregiving and improved caregiver well-being. However, it may have disproportionately benefited caregivers of higher socioeconomic status, potentially exacerbating disparities in caregiver stress. Future policies should aim to mitigate this unintended consequence.

## Key Points

**Question:**

Was the Balancing Incentives Program, designed under the US Patient Protection and Affordable Care Act to shift long-term care out of institutions and into the home, associated with changes in the prevalence and frequency of informal caregiving or changes in caregiver well-being from 2011 to 2018?

**Findings:**

In this cohort study of 38 343 participants, the Balancing Incentives Program was associated with increased daily caregiving and improvements in caregiver sleep, a marker of well-being. These benefits accrued mainly to caregivers of higher socioeconomic status.

**Meaning:**

These findings were consistent with the goals of the Balancing Incentives Program but suggest disparities in caregiver stress by socioeconomic status.

## Teaser

This cohort study assessed the association of the US Patient Protection and Affordable Care Act (ACA) Balancing Incentives Program with the prevalence and frequency of informal caregiving in the US from 2011 to 2018.

## Introduction

Spending on long-term services and supports (LTSS) for people with functional limitations is projected to increase substantially over the coming decades in the US.^1^ To address this growth, recent policies have incentivized states to shift LTSS out of institutional settings and into homes. One such policy is the Patient Protection and Affordable Care Act Balancing Incentives Program (BIP), which provided $2.4 billion of enhanced Medicaid matching funds between 2011 and 2015 to states that made dedicated changes to their long-term care enrollment processes to encourage care delivery in the home and in community-based settings.

States that spent greater than 50% of their 2009 Medicaid LTSS budget on home and community-based services were ineligible to participate in the BIP.^2^ Of the 38 states eligible to participate, 18 states chose to participate and 20 states were eligible but did not participate (Figure).^3^ Implementation timing varied among participating states, with start dates ranging from April 2012 to April 2016. Although states that implemented the BIP were given standard expenditure and infrastructure goals, they had broad discretion with respect to the specific activities, programs, and populations on which they chose to focus their efforts. Some states also adopted secondary, voluntary goals as part of the program. Although states had the option to increase home and community-based LTSS spending by either increasing expenditures among households already being served or expanding the number of people receiving services, most states focused on the latter (eTable 1 in the Supplement).^4^ State-level program evaluations have focused on whether broad spending goals were met and whether states were able to implement core programs. Results from these evaluations have broadly found that although some states struggled to meet targets, they were largely successful in expanding home and community-based supports and increasing spending on these services.

**Figure.**
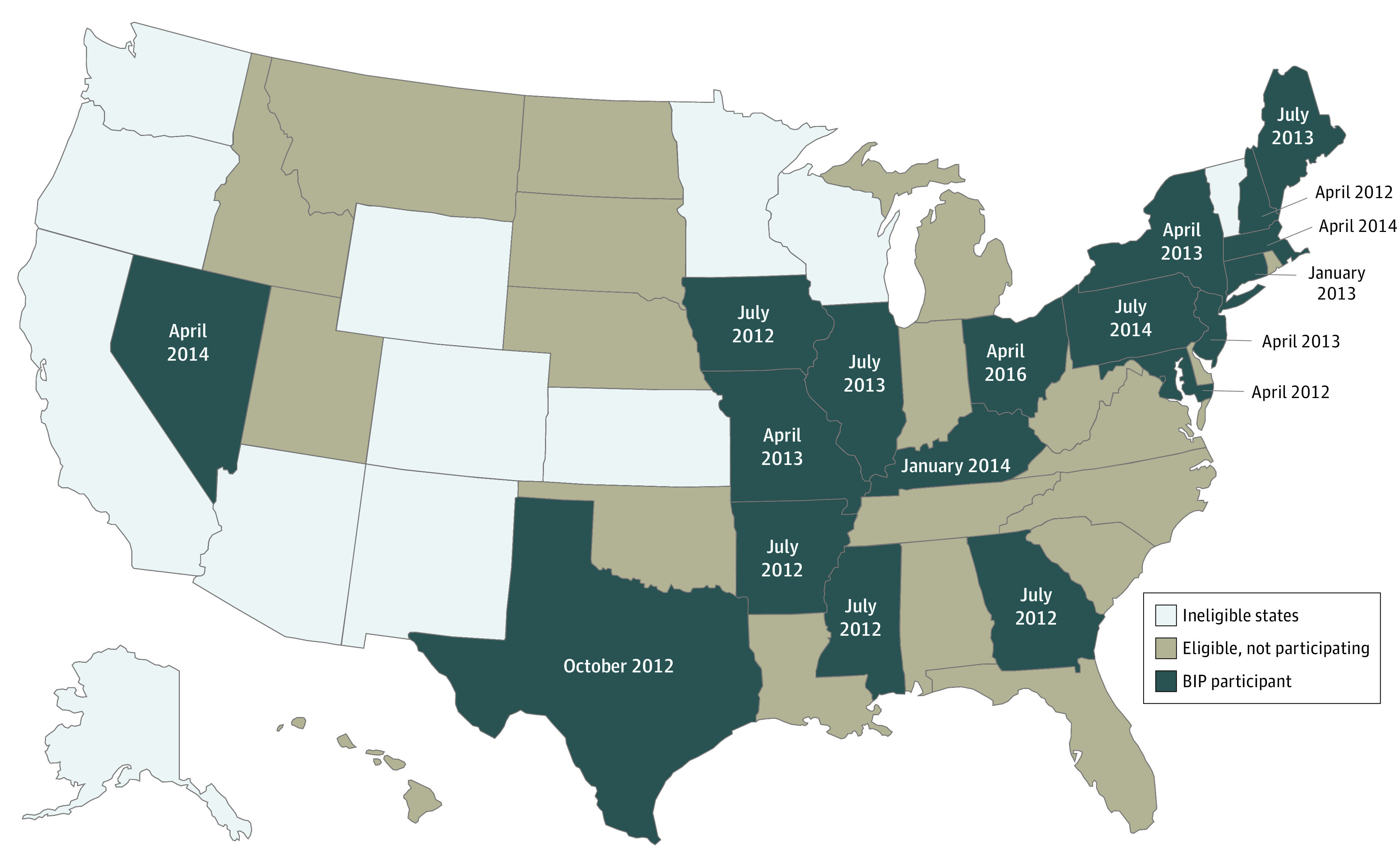
State Eligibility for and Participation in the Patient Protection and Affordable Care Act Balancing Incentives Program (BIP)

Understanding of the consequences of this expansion for informal caregivers is a critical priority, especially as federal and state programs continue to promote more home-based care. Although shifting care out of institutional settings may decrease spending, it could also increase the demands placed on the approximately 34.2 million people who serve as informal caregivers for adult family members or friends.^5^ Informal caregivers often fulfill their roles at physical and psychological costs^6^; if care responsibilities increased after implementation of the BIP, such costs may have been exacerbated. On the other hand, resources allocated under the BIP may have provided additional supports for home- and community-based services, which could have mitigated some of the stress experienced by informal caregivers, particularly for those who may have had easier access to such services.

In this study, we examined the association between the BIP and informal caregiving activities in the US. Using a difference-in-differences (DID) approach, we compared changes in the prevalence and frequency of caregiving activities in states that participated in the BIP compared with states that did not.

## Methods

### Data and Sample

This cohort study used data from the American Time Use Survey (ATUS), an annual telephone-based survey in the US, the primary purpose of which is to develop nationally representative estimates of how people spend their time. The survey collects detailed data on the activities of respondents over a single survey day preceding data collection. Activities reported by the ATUS include various personal care and household activities, eating and drinking, working, communicating, and other community and religious activities. Starting in 2011, the ATUS began collecting data on caregiving activities. These data have been used in prior contexts to evaluate caregiving activities in the US.^7^ The ATUS also collects respondent characteristics of self-reported age, sex, race/ethnicity, educational level, employment status, income, citizenship status, and household size and data on the respondent relationship to and age of care recipients. This study was deemed exempt from institutional review board of the University of Pennsylvania owing to the use of publicly available data. This study followed the Strengthening the Reporting of Observational Studies in Epidemiology (STROBE) reporting guideline.^8^

Within the ATUS data, we defined 2 samples of respondents: all respondents and the subset who identified as caregivers. Caregivers were defined as any respondent older than 18 who indicated “yes” as to whether they had provided care to an elderly relative or friend in the past 3 months.

### Exposure

The primary exposure was a time-varying, state-level indicator of BIP participation obtained from publicly available resources. We excluded states that were ineligible to participate in the BIP based on 2009 Medicaid LTSS spending.^2^ States that participated in the BIP were categorized as non–BIP-adopting states before the time of program implementation (defined as 6 months after formally adopting the BIP) and BIP-adopting states after implementation. All other eligible states were categorized as non–BIP-adopting states throughout the study period.

### Outcomes

The primary outcomes included the prevalence and frequency of caregiving activities. For the prevalence of caregiving, we used the sample of all respondents and examined changes in the number of individuals responding “yes” to the question regarding providing care for an elderly relative or friend in the past 3 months. A priori, we hypothesized that the prevalence of caregiving would increase in BIP-adopting states, in line with one of the stated goals of the program to shift LTSS out of institutional settings and into homes. Caregiving frequency was assessed based on how often caregivers provided daily care. We used the survey question asking how often caregivers provided care to elderly relatives of friends to create a binary marker for whether a caregiver provided daily care or less than daily care. From this binary marker, we created an outcome reflecting the proportion of caregivers providing daily care. We also hypothesized that daily caregiving would increase more in BIP-adopting states than non–BIP-adopting states as caregiving activities were shifted into the home.

Our secondary outcome was minutes of sleep, assessed among the sample of caregivers. We chose this measure because sleep is a known marker of caregiver well-being^9^ and the ATUS has been used in other contexts to examine changes in sleep.^10^ Prior studies have demonstrated a positive association between sleep and the mental health of caregivers.^11^ Improved sleep is also associated with well-being on scales that measure levels of activity restriction and pleasurable activities for caregivers.^12^ More generally, sleep has been correlated with individual health outcomes and well-being in multiple studies.^13^ Although the ATUS collects information on many daily activities, there was insufficient reporting to allow for meaningful analysis of other potential markers of well-being. Of importance, the ATUS only captures activity data for the 24 hours before survey administration; thus, sleep was the only marker of well-being that could be evaluated with sufficient statistical power for all survey participants.

### Statistical Analysis

First, we described the following respondent and caregiver characteristics in the BIP-adopting and non–BIP-adopting states: age, race/ethnicity, and employment status as well as the nature and age of their relationship with care recipients. We then performed a DID analysis using linear regression to examine the association between state-level participation in the BIP and our primary and secondary outcomes, using the sample of all respondents and the sample of caregivers, respectively.^14^ We adjusted for state and year fixed effects to account for secular trends and time invariant factors across states during the study period, respondent characteristics (age, sex, race/ethnicity, educational level, employment status, income, citizenship status, and household size), and survey timing (day of the week, month, and holiday status). All analyses used survey weights provided by the ATUS.

Given prior work showing that caregiving responsibilities and experiences differ by socioeconomic status,^15^ we performed exploratory, post hoc analyses stratifying the caregiver sample by household income (<$60 000 annually or≥$60 000), employment status (unemployed, employed part-time, or employed full-time), and educational level (high school or less, some secondary school, or bachelor’s degree or more). We tested each outcome for parallel trends between BIP-adopting and non–BIP-adopting states in the years before implementation.

For all analyses, we clustered SEs at the state level and considered *P* < .05 to be statistically significant using two-tailed tests. Stata, version 15.1 (StataCorp LLC) was used for analysis.

## Results

We identified 38 343 respondents in BIP-adopting states (median [IQR] age of 47 (31-61), 51.9% female, 15.0% Black, 14.5% Hispanic, 50.8% employed full time) and 26 437 respondents in non–BIP-adopting states (median age, 48 years [interquartile range (IQR), 32-62 years]; 52.7% female; 15.0% Black; 9.7% Hispanic; 49.2% employed full time) (Table 1 and eFigure in the Supplement) When we limited the sample to caregivers, there were 7428 in BIP-adopting states (median age, 51 years [IQR, 37-61 years]; 55.6% female; 13.5% Black; 9.4% Hispanic; 49.8% employed full time) and 5527 in non–BIP-adopting states (median age, 52 years [IQR, 38-62 years]; 57.9% female; 14.2% Black; 5.7% Hispanic; 46.9% employed full time). Parents and parents-in-law were the most frequent recipients of care in both BIP-adopting and non–BIP-adopting states (44.6% and 43.0% of care recipients, respectively), followed by friends and neighbors (16.1% and 18.6%, respectively), other nonrelatives (14.4% and 12.7%, respectively), grandparents and great-grandparents (10.9 and 10.6%, respectively), other relatives (9.7% and 9.8%, respectively), and spouses or partners (4.3% and 5.3%, respectively). The median age of care recipients was 79 years (IQR, 70-85 years) in both BIP-adopting and non–BIP-adopting states.

**Table 1.** Characteristics of All Respondents, Caregivers, and Recipients of Care in BIP-Adopting and Non–BIP-Adopting States^a^

Characteristic	All respondents		Caregivers only	
	BIP-adopting states (n = 38 343)	Non–BIP-adopting states (n = 26 437)	BIP-adopting states (n = 7428)	Non–BIP-adopting states (n = 5527)
Age, median (IQR), y				
Caregiver	47 (31-61)	48 (32-62)	51 (37-61)	52 (38-62)
Recipient of care	NA	NA	79 (15)	79 (15)
Female	51.9	52.7	55.6	57.9
Race				
Black	14.2	15.0	13.5	14.2
White	80.1	80.0	82.7	82.4
Other	5.7	5.0	3.8	3.4
Hispanic ethnicity	14.5	9.7	9.4	5.7
Employed				
Part time	13.0	13.7	14.6	15.2
Full time	50.8	49.2	49.8	46.9
Relationship to care recipient				
Parent or parent-in-law	NA	NA	44.6	43.0
Spouse or partner	NA	NA	4.3	5.3
Grandparent or great-grandparent	NA	NA	10.9	10.6
Friend or neighbor	NA	NA	16.1	18.6
Other relative	NA	NA	9.7	9.8
Other nonrelative or not defined	NA	NA	14.4	12.7

Abbreviations: BIP, Balancing Incentive Program; IQR, interquartile range; NA, not applicable.

^a^ Data are presented as percentages of respondents, unless otherwise indicated. Sample sizes are based on unweighted analysis, and summary statistics reflect survey weights.

For our primary outcomes, we found no significant difference in the prevalence of caregiving between BIP-adopting and non–BIP-adopting states after participation in the program (DID, 0.00%; 95%, −0.01% to 0.01%; *P* = .94) (Table 2). In the sample of caregivers, 19.6% of those in BIP-adopting states and 20.9% in non–BIP-adopting states provided daily care over the entire study period (eTable 2 in the Supplement gives unweighted values). Implementation of the BIP was associated with a 3.2% (95% CI, 0.3%-6.0%; *P* = .03) increase in the percentage of caregivers providing daily care compared with non–BIP-adopting states. For our secondary outcome, the mean (SD) time spent sleeping for caregivers over the entire study period was 517.9 (134.6) minutes in BIP-adopting states and 520.6 (135.3) minutes in non–BIP-adopting states (eTable 2 in the Supplement gives unweighted values). Caregivers in BIP-adopting states reported an increase in daily time sleeping of 15.6 minutes (95% CI, 4.9-26.2 minutes; *P* = .005) associated with program implementation compared with those in non–BIP-adopting states.

**Table 2.** Difference-in-Differences Estimates for Primary and Secondary Outcomes

Outcome	Change in outcome associated with BIP intervention (95% CI)	*P* value
Primary: caregiving prevalence and frequency, %		
Likelihood of respondent being a caregiver	0.0 (−0.01 to 0.01)	.94
Share of caregivers providing daily care	3.2 (0.3 to 6.0)	.03
Secondary: minutes of sleep		
Time slept	15.6 (4.9 to 26.2)	.005

Abbreviation: BIP, Balancing Incentive Program.

In stratified analyses, caregivers with a high school education or less provided daily care more often in BIP-adopting states compared with non–BIP-adopting states after program implementation (DID, 6.8%; 95% CI, 2.0%-11.7%; *P* = .007) (Table 3). Caregivers in BIP-adopting states also reported more sleep if they had annual household incomes greater than or equal to $60 000 (DID, 16.9 minutes; 95% CI, 5.9-27.9 minutes; *P* = .004), worked full time (DID, 15.3 minutes; 95% CI, 2.9-27.8 minutes; *P* = .02), had some secondary education (DID, 25.1 minutes; 95% CI, 6.5-43.8 minutes; *P* = .01) or had a bachelor’s degree (DID, 19.9 minutes; 95% CI, 4.1-35.8 minutes; *P* = .02). After BIP implementation, daily caregivers in BIP-adopting states reported an additional 28.1 minutes (95% CI, 9.2-47.0 minutes; *P* = .005) of sleep compared with those in non–BIP-adopting states. For all outcomes, we found no evidence to reject the assumption of parallel trends (eTable 3 in the Supplement).

**Table 3.** Difference-in-Differences Estimates for Secondary Outcomes by Caregiver Subgroups

Characteristic	Frequency of care			Sleep		
	Respondents, No.	Change in caregivers reporting daily care, DID estimate, % (95% CI)	*P* value	Respondents, No.	Change in time spent sleeping, DID estimate, minutes (95% CI)	*P* value
Annual household income, $						
<60 000	7120	2.8 (−2.2 to 7.8)	.26	7140	13.6 (−3.9 to 31.2)	.12
≥60 000	5805	3.7 (0.4 to 6.9)	.03	5815	16.9 (5.9 to 27.9)	.004
Employment status						
Not employed	4835	3.0 (−2.1 to 8.2)	.23	4847	15.1 (−5.7 to 35.9)	.15
Part time	1786	0.3 (−8.6 to 9.2)	.94	1792	16.2 (−11.6 to 44.1)	.25
Full time	6304	3.1 (−0.2 to 6.5)	.06	6316	15.3 (2.9 to 27.8)	.02
Educational level						
High school or less	3993	6.8 (2.0 to 11.7)	.007	4010	3.3 (−13.2 to 19.9)	.68
Some secondary	3891	−0.8 (−6.2 to 4.6)	.76	3894	25.1 (6.5 to 43.8)	.01
Bachelor’s degre or greater	5041	2.4 (−1.5 to 6.3)	.23	5051	19.9 (4.1 to 35.8)	.02
Frequency of caregiving						
Less than daily	NA	NA	NA	10 616	12.4 (1.5 to 23.3)	.03
Daily	NA	NA	NA	2309	28.1 (9.2 to 47.0)	.005

Abbreviation: DID, difference-in-differences; NA, not applicable.

## Discussion

The BIP was designed to shift care for people with functional limitations from institutions to the home and community-based settings where informal caregivers are likely to assume the primary responsibility for care. We found that BIP was not associated with an increase in the overall prevalence of caregiving but was associated with an increase in daily caregiving activities among existing caregivers. Increased caregiving activities may be associated with exacerbation of baseline stressors; however, caregivers in BIP-adopting states reported increases in sleep, a known measure of caregiver well-being, compared with caregivers in non–BIP-adopting states. Of importance, the increase in sleep only accrued to caregivers with higher socioeconomic status.

Policies designed to divert LTSS from institutions to homes likely have multidirectional effects on caregivers and caregiving activities. Although the BIP was associated with more daily caregiving activities, our finding of more sleep after BIP implementation suggests that the program may have offset some of the personal costs of more frequent care. However, the BIP may have not been associated with reduced stress among caregivers with lower socioeconomic status. This is consistent with existing caregiving literature,^16^ which has demonstrated greater caregiving intensity among non-White caregivers with lower socioeconomic status.

There are several potential explanations for these findings. The provision of long-term care at home may be more difficult among lower-income seniors, who face multiple challenges associated with social determinants of health. For example, housing instability, the need for home repairs, and pest remediation are more likely to complicate the provision of home-based care and increase the stress experienced by informal caregivers. Lower-income seniors may also have reduced access to home- and community-based services because of limited transportation, geographic isolation, or lower levels of health literacy despite a potentially greater need for LTSS overall.^17^ In contrast, caregivers with higher socioeconomic status and educational level may be better positioned to use existing resources or have the ability to pay for additional home care out of pocket. Individuals for whom they care are also more likely to have long-term care insurance to cover the cost of additional home care hours, further reducing the strain of caregiving among higher-income caregivers.^18^

Taken together, these findings have several policy implications. As policy makers continue to focus on shifting LTSS spending out of institutional settings and into the home, a one-size-fits-all approach may risk exacerbating preexisting disparities and further stressing already at-risk low-income caregivers. Future policies could mitigate some of these consequences by designing programs that focus specifically on low-income caregivers. Such programs could address the increased need for nonmedical services, such as home repairs or affordable housing options among low-income care recipients. Although the BIP offered a broad federal policy that gave states marked flexibility in its design and implementation, more directive federal policies may also be necessary to ensure that future efforts can mitigate underlying disparities among caregivers.

### Limitations

This study has limitations. First, our secondary outcome, minutes of caregiver sleep, is not a direct measure of caregiver well-being and can only be interpreted cautiously as a marker of it. Prior work has shown that caregiver stress manifests in multiple ways, including poor health outcomes and increased psychosocial and financial stress^6^; however, our use of survey data precluded our ability to assess these other dimensions. Of importance, sleep has been shown to be a marker of well-being given its association with multiple medical comorbidities, mental health, and quality of life.^13^ Second, we cannot attribute causal effects to BIP implementation. However, our DID approach accounts for secular trends in nonparticipating states and also takes advantage of the staggered implementation of the BIP across states, making false attribution of the effects to BIP less likely. Third, owing to a lack of data, we were unable to account for certain characteristics of caregivers, care recipients, or caregiving activities, such as the location of care provided (eg, in a skilled nursing facility vs at home), availability of additional paid caregivers in the home, or other family members providing care. Fourth, there is marked heterogeneity in the implementation of the BIP program across states for which our models did not account. Our analysis was underpowered to assess these differences. Nonetheless, the overarching goals of the program remained the same across states, and we found multiple shared themes among BIP-adopting states.

## Conclusions

In this cohort study, the BIP was associated with increased daily caregiving and improved caregiver well-being; however, it may have disproportionately benefited caregivers of higher socioeconomic status, potentially increasing disparities in caregiver stress. Future policies should aim to mitigate this unintended consequence by providing additional support and home-based services to at-risk caregiver populations.

## Notes

Supplement. **eTable 1.** Summary of Timing, Funding, BIP Goal Achievement, and Strategies used by BIP Participating States Through June 2017 **eFigure.** Definition of two study samples **eTable 2.** Mean weighted and unweighted values for primary and secondary outcomes **eTable 3.** Test of pre-trends for outcomes
