# Supplementary material for: Prevalence of Informal Caregiving in States Participating in the US Patient Protection and Affordable Care Act Balancing Incentive Program, 2011-2018
Source: JAMA Netw Open. 2020 Dec 15;3(12):e2025833. doi: 10.1001/jamanetworkopen.2020.25833 (PMC7739120; doi:10.1001/jamanetworkopen.2020.25833)
Supplement: Supplement. — eTable 1. Summary of Timing, Funding, BIP Goal Achievement, and Strategies used by BIP Participating States Through June 2017 eFigure. Definition of two study samples eTable 2. Mean weighted and unweighted values for primary and secondary outcomes eTable 3. Test of pre-trends for outcomes [file jamanetwopen-e2025833-s001.pdf]

Supplemental Online Content

Anastos-Wallen R, Werner RM, Chatterjee P. Prevalence of informal caregiving in states participating in the US Patient Protection and Affordable Care Act Balancing Incentive Program, 2011-2018. *JAMA Netw Open.* 2020;3(12):e2025833. doi:10.1001/jamanetworkopen.2020.25833

eTable 1. Summary of Timing, Funding, BIP Goal Achievement, and Strategies used by BIP Participating States Through June 2017
eFigure. Definition of two study samples
eTable 2. Mean weighted and unweighted values for primary and secondary outcomes
eTable 3. Test of pre-trends for outcomes

This supplemental material has been provided by the authors to give readers additional information about their work.

© 2020 Anastos-Wallen R et al. *JAMA Network Open.*

eTable 1: Summary of Timing, Funding, BIP Goal Achievement, and Strategies used by BIP Participating States Through June 2017

	Summary of Timing, Funding, BIP Goal Achievement, and Strategies used by BIP Participating States Through June 2017												
	Timing	Funding	Status with respect to core BIP goals			Strategies used to expand HCBS spending as share of all LTSS expenditures		Strategies used to increase the number of people receiving HBCS					
	Start date	Extra funding earned ($M)	No wrong door / Single Entry Point	Conflict free case management	Develop Core Standardized Assessment	Increase payment rates	Increase services to current recipients	Expand mental health services	Increase support of transitions of care	Target new populations	Reduce HCBS waitlists	Increase HCBS waiver slots	Other
**Arkansas**	Apr 2013	40.4	Yes	Yes	Yes	X							X
**Connecticut**	Jan 2013	78.1	Yes	Yes	Yes	X	X	X	X	X		X	
**Georgia**	Jul 2012	76.2	Yes	Yes	Yes	X	X	X				X	X
**Illinois**	Jul 2013	96.2	No	Yes	Yes	X	X	X	X	X	X	X	
**Iowa**	Jul 2012	63.7	Yes	Yes	Yes	X		X		X		X	
**Kentucky**	Jan 2014	27.3	Yes	Yes	Yes							X	
**Maine**	Jul 2013	21.1	Yes	Yes	Yes							X	
**Maryland**	Apr 2012	106.7	Yes	Yes	Yes	X	X	X	X	X	X	X	
**Massachusetts**	Apr 2014	119.5	Yes	Yes	Yes	X	X	X					
**Mississippi**	Jul 2012	74.7	Yes	Yes	Yes	X	X	X		X	X	X	
**Missouri**	Jul 2012	110.5	Yes	Yes	Yes					X		X	
**Nevada**	Apr 2014	7.2	No	Yes	Yes				X	X			
**New Hampshire**	Apr 2012	28.3	Yes	Yes	Yes	X							X
**New Jersey**	Apr 2013	100.6	Yes	Yes	Yes	X	X	X	X	X			X
**New York**	Apr 2013	619.3	Yes	Yes	Yes		X						X
**Ohio**	Apr 2016	165.8	No	Yes	Yes		X	X		X		X	
**Pennsylvania**	Jul 2014	104.2	No	Yes	Yes							X	
**Texas**	Oct 2012	284.5	Yes	Yes	Yes	X	X	X		X	X	X	

Source: https://aspe.hhs.gov/basic-report/final-process-evaluation-balancing-incentive-program, https://www.balancingincentiveprogram.org/bip-glance?location=oh

© 2020 Anastos-Wallen R et al. *JAMA Network Open.*

eFigure: Definition of two study samples

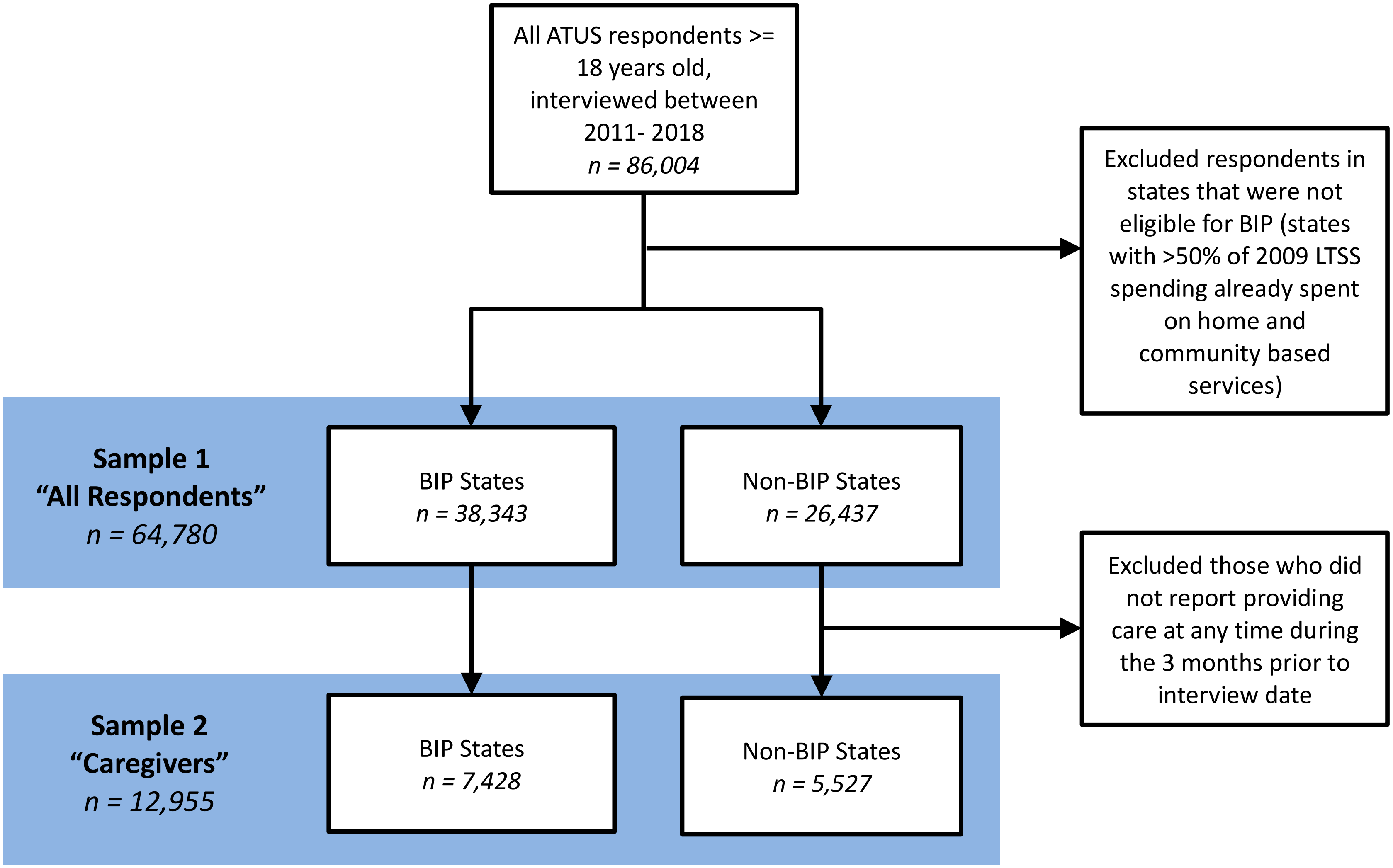

© 2020 Anastos-Wallen R et al. *JAMA Network Open.*

eTable 2: Mean weighted and unweighted values for primary and secondary outcomes

Mean weighted and unweighted values for primary and secondary outcomes in data					
		Unweighted Values		Weighted Values	
	**Mean (SD) for outcome**	**BIP States**	**Non-BIP States**	**BIP States**	**Non-BIP States**
**Primary Outcomes:** Caregiving prevalence and frequency	Percent of respondents who identify as caregiver	19.7% (39.8%)	21.3 (40.9%)	20.2% (40.1%)	21.7% (41.2%)
	Percent of caregivers who provide daily care	17.1% (37.7%)	18.8% (39.1%)	19.6% (39.7%)	20.9% (40.6%)
**Secondary Outcome:** Minutes of sleep	Time slept in minutes	525.3 (137.2)	527.2 (137.4)	517.9 (134.6)	520.6 (135.3)

© 2020 Anastos-Wallen R et al. *JAMA Network Open.*

eTable 3: test of pre-trends for outcomes

Test of pre-trends trends for outcomes			
		Differences-in-Differences co-efficient	**P**
**Primary Outcomes:** Caregiving prevalence and frequency	Likelihood of respondent being a caregiver	0.0%	0.82
	Share of caregivers providing daily care	0.4%	0.41
**Secondary Outcome:** Minutes of sleep	Time slept (minutes)	2.1	0.16

© 2020 Anastos-Wallen R et al. *JAMA Network Open.*
